# Are Food Additives a Really Problematic Hidden Source of Potassium for Chronic Kidney Disease Patients?

**DOI:** 10.3390/nu13103569

**Published:** 2021-10-12

**Authors:** Montserrat Martínez-Pineda, Antonio Vercet, Cristina Yagüe-Ruiz

**Affiliations:** Faculty of Health and Sports Science, University of Zaragoza, 22002 Huesca, Spain; vercet@unizar.es (A.V.); cyague@unizar.es (C.Y.-R.)

**Keywords:** additives, potassium, chronic kidney disease, CKD, processed food

## Abstract

Dietary treatment in chronic kidney disease (CKD) recommends limiting the consumption of foods rich in potassium to reduce risk of hyperkalemia. Currently, the increased supply of processed foods on the market could be a new “hidden” source of potassium for these patients, which is causing concern among health professionals who treat them. The aim of this study was to check which EU authorized food additives contain potassium, its conditions of use and classified them according to their risk for CKD patients. In addition, the frequency of appearance of potassium additives in processed foods in a European sample through the analysis of 715 products labeling from France, Germany, and Spain were evaluated. Results showed 41 potassium-containing additives allowed in the European Union, but only 16 were identified, being the most frequent: E202; E252, E340, E450, E452, E508, and E950. The 37.6% of the processed products analyzed contained at least one potassium additive. The food categories that showed the greatest presence of additives were breaded products, meat derivatives, non-alcoholic beverage, ready-to-eat products, and cereal derivatives. Potassium additives are widely distributed in processed foods and therefore pose a risk of hidden sources of potassium in CKD dietary management. These results could be really useful for developing educational tools for CKD patients.

## 1. Introduction

The global all-age chronic kidney disease (CKD) prevalence has increased 29.3% and mortality rate 41.5% in the last three decades [1]. According the 2020 Kidney Disease Outcome Quality Initiative (K/DOQI), the management of CKD should be focused not only in pharmacological treatment, also in nutritional and dietary interventions [2]. The aim of dietary and nutritional management in CKD is the maintenance of an optimal nutritional status, while preventing common complications of chronic renal failure in order to delay other treatments such as dialysis [3]. Kidney is the organ responsible for potassium regulation in the body so that the risk of hyperkalemia increases as kidney function declines and CKD progresses. The prevalence of this metabolic abnormality range between 10–16% depending on the disease stage and renal replacement therapy [4], and has been related to an increase in the risk of mortality in CKD patients [5].

It has been observed that in the early stages of CKD higher potassium intake is not so harmful, and even seems to have a protective effect against CV disease and the progression of renal disease [6]. However, there is some controversy in advanced stages of CKD and no consensus in the total dietary amount limit. Scientific societies currently recommend restrictions in dietary potassium intake, especially in late CKD (stage 3–5), adjusting it on a patient’s individual situation [2,3,7].

On this matter, CKD dietary guidelines recommend minimizing naturally high potassium content food intake, such as some fruits, vegetables, legumes, and nuts. Potassium reduction through culinary treatments, like soaking or boiling, are also recommended. Furthermore, in the last years, food additives have been included as a new disturbing agent in CKD diet management [8].

Food additives are defined as “any substance not normally consumed as a food in itself and not normally used as a characteristic ingredient of food, whether or not it has nutritive value”. Its addition to food for technological purposes, such as preservation, coloring, sweetening, etc. during the preparation of food has the result that food additives become a final component of the food [9]. It has been observed that inorganic phosphate salts present in additives are almost completely absorbed while organic phosphorus present naturally in foods has lower bioavailability [10]. Educational interventions in end-stage renal disease patients to avoid phosphorus-containing food additives improved serum phosphorus levels [11,12]. However, the identification of phosphorus-containing additives through labeling as an educational goal has been also highlighted [13].

The limited current evidence suggests a similar behavior of additives and salts with potassium, the bioavailability of which appears to be near the 100% [14,15]. Therefore, wider depth knowledge of potassium additives, as well as food-containing these additives is crucial for the dietary management of CKD patients and the development of educational tools and material to prevent hyperkalemia in these patients.

The aim of this study was to analyze which EU authorized food additives contain potassium, in which foods its inclusion is allowed and the maximum level permitted. In addition, the frequency of appearance of these additives in processed foods in European Union through the analysis of their labeling was evaluated.

## 2. Materials and Methods

### 2.1. Analysis of Current European Food Additives Legislation

A detailed analysis of the current legislation on authorized additives at European level was carried out, specifically Commission Regulation (EU) no.1129/2011 [16] amending Annex II of the Regulation (CE) no.1333/2008 of the European Parliament and of the Council to establish a list of food additives of the Union [17], and subsequent modifications.

From it the following information was obtained:

(1) What food additives contain potassium in their chemical formulation.
(2) In which food categories are each of them allowed.
(3) The maximum level of each additive allowed according to food categories.

### 2.2. Potassium Content in Potassium-Additives

Based on the data obtained from the previous analysis, the real relevance with respect to the weight of potassium in the chemical formula of the additive molecule was evaluated.

For this, firstly, the chemical structure, molecular weight and purity of each potassium additive were obtained from Regulation (EU) no.231/2012 [18] and subsequent modifications. Then, the proportion, by weight, of the potassium atom (or atoms) in the molecular weight of the additive was calculated. It was also taken into account the purity degree of each additive usually found in the market. Finally, all potassium additives were classified into three groups: (i) additives with low potassium content (LKC) if potassium represented <25% of the molecule weight, (ii) additives with moderate potassium content (MKC) if potassium weight represented between 25 and 40% of the total molecule weight and (iii) high potassium content (HKC) additives when the weight of potassium was 40% or more of the additive total weight.

### 2.3. Label Analysis

A cross-sectional design was utilized to examine the presence and frequency of appearance of potassium additives in three European countries: France, Germany, and Spain. We analyzed the labeling of processed products belonging to the food categories in which the additives selected for the study were authorized. Twelve food categories were analyzed: dairy products and derivatives, ice creams, cereals and derivatives, fruit/vegetables and derivatives, meat derivatives, fish-seafood and derivatives, non-alcoholic beverages, sauces, snacks and confectionery, breaded products, ready-to-eat foods and vegan products. Breaded products, ready-to-eat foods, and vegan products categories are not specifically classified in Commission Regulation (EU) no.1129/2011 [16]. However, its inclusion in the analysis is relevant since these groups englobe products made of mixed ingredients that may contain potassium additives. A total of 715 product labelling were analyzed (Table 1). For each kind of product (ex. melted cheese), a minimum of three different commercial brands were analyzed. All products selected had suffered any type of technological process and were susceptible to contain potassium additives according to the European legislation on additives. When possible, homologous products from the three countries were included in the analysis. Likewise, an attempt was made to include typical and frequently consumed products from each country.

At least three different large grocery store chains in each country were scanned between March–July 2021. The grocery store chains scanned were: Carrefour (Carrefour Groupe, Massy, France), Auchan (Auchan Groupe, Croix, France), Monoprix (Monoprix SA., Clichy, France) in France, Edeka (Edeka Stiftung & Co. KG, Hamburg, Germany), Rewe (Rewe GmbH, Köln, Germany), Norma (Norma GmbH, Fürth, Germany), Lidl (Lidl Stiftung & Co. KG, Neckarsulm, Germany), Aldi (Aldi Sür SA, Mülheim, Germany) in Germany, and Carrefour (Carrefour SA, Madrid, Spain), Alcampo (Alcampo, SA, Madrid, Spain), Mercadona (Mercadona, SA., Tavernes Blanques-Valencia, Spain) in Spain, respectively. Both generic and named brand products were included in the analysis. During labelling analysis, the presence and absence of potassium additives were noted in each product, as well as the technological use made of them indicated in each case. Subsequently, an analysis of frequency appearance was carried out by type of additive and technological use, as well as by food category, so that a relationship between them could be established.

### 2.4. Statistical Analysis

Frequency of appearance and statistical analyses were performed with Microsoft Excel software (Microsoft Corporation, Redmond, WA, USA).

## 3. Results

### 3.1. Authorized Additives with Potassium

According to current legislation, of all the food additives authorized in Europe, 41 of them present potassium in their formulation (Table 2). Based on additive purity degree, its molecular weight, and the proportional weight of the potassium atoms in it, foods were classified into three groups: additives with low potassium content (LKC) (<25% by weight of potassium), with moderate potassium content (MKC) (25–39% by weight of potassium) and high potassium content (HKC) (≥40% by weight of potassium).

The results showed in Table 2 that of the 41 additives with potassium in their formulation, 44% belonged to LKC group, 37% to MKC group and 19% to HKC group. The additive with the highest proportion of potassium in its formulation, 62.7%, is E525-Potassium hydroxide, which in the industry is mainly used as an acidity regulator.

### 3.2. Food Categories and Maximum Level Authorized

To examine the potential danger for CKD patients posed by hidden potassium in foods derivate from additives, in addition to knowing which additives contain potassium in their formulation, it is necessary to know in which foods they can be used and the maximum level allowed for each.

The results of this analysis showed that of the 8 additives with a high potassium content in their formulation (HKC), half belonged to Group I according to Commission Regulation (EU) No. 1129/2011 [16], which means they could be added to a wide range variety of foods (Table 3), in *Quantum satis* quantities. This implies that according to current legislation, no maximum numerical level is specified and substances shall be used in accordance with good manufacturing practice, at a level not higher than is necessary to achieve the intended purpose and provided the consumer is not misled. The HKC additives that are regulated under this criterion are: potassium hydrogen carbonate (E501ii), potassium chloride (E508), potassium sulfate (E515) and potassium hydroxide (E525) mentioned above (Table 2).

The remaining HKC group additives, potassium nitrite (E249), di- and tripotassium phosphate (E340), and tetrapotassium diphosphate (E450), have a more restricted use and maximum levels are established for certain foods. Specifically, the last two (E340 and E450) are usually considered together by legislation and regulated as one group (E338-452: Phosphoric acid, phosphates, di-, tri- and polyphosphates) together with other salts of phosphoric acid such as calcium or sodium. Its use is allowed in a wide variety of foods and maximum levels allowed range from 1000 to 50,000 mg/kg or L, and even in some specific cases such as *Quantum satis* (Appendix A). It should be noted that its great variety of technological functions such as acidulants, acidity regulators, melting salts, raising agents, and stabilizers, make them very susceptible to being used and therefore be present a large number of products.

Regarding potassium nitrite (E249), together with sodium nitrite (E250), current legislation allowed it use mainly in processed meats, both heat-treated and not heat-treated in amounts between 100 and 150 mg/kg.

However, it is also relevant to take into account that some of the additives belonging to MKC group are also included in legislation as Group I according to Commission Regulation (EU) No. 1129/2011, so that can be used under *Quantum satis* criteria into a very wide variety of foods, which makes it interesting to follow up. These are specifically the following additives: tripotassium citrate (E332), dipotassium tartrate (E336) and potassium malate (E351).

Finally, within the MKC group of additives, it is worth highlighting potassium sorbate (E202) and potassium nitrate (E252). The main technological use of these additives is a preservative function and their use is very common in the food industry. Although the maximum level allowed in food are regulated by Commission Regulation (EU) No. 1129/2011 [16], it varies between 200 mg/kg and *Quantum satis* for potassium sorbate (E202) and 150 to 500 mg/kg in potassium nitrate (E252). Moreover, its use is allowed in a wide variety of products, especially potassium sorbate (E202) (Appendix A—Table A1 and Table A2), so it is necessary to consider them as probable hidden sources of potassium in food.

### 3.3. Potassium Additives in Food

**Figure 1:**
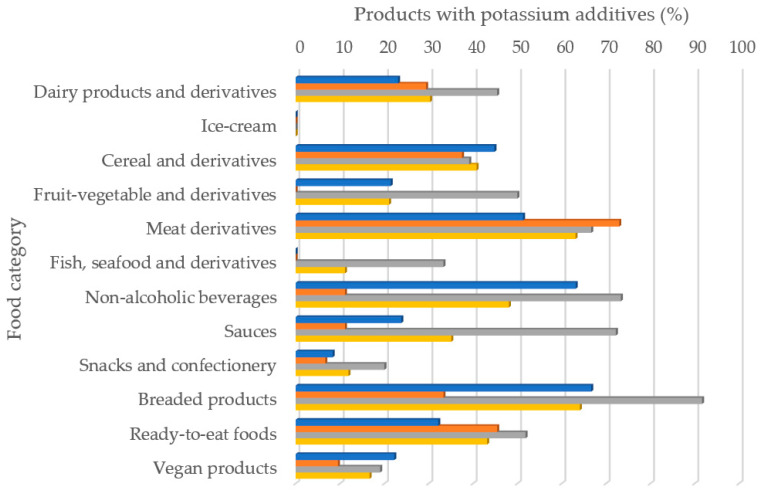
Percentage of foods with some type of potassium additive according to food category. 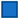 France; 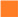 Germany; 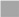 Spain; 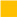 Global sample.

The results of labeling analysis, including the presence/absence, type of additive and technological function of all the products analyzed are shown. Of the 715 food products analyzed, 269 contained some additive with potassium in their formulation, which represents 37.6%. It was observed important differences between countries. Spain was the country with more presence of potassium additives in their products (50%), followed by France (34.1%) and then Germany (30.5%). Figure 1 shows the percentage of foods that had some type of additive with potassium content in their formulation according to food category. All food categories showed potassium additives in its ingredients lists with the exception of ice-cream in all countries, as well as, fish derivatives in France and Germany, and fruit/vegetable derivatives in Germany. As an average, in all studied samples, the products that presented the greatest presence of potassium additives among its ingredients were breaded products and meat derivatives, followed by non-alcoholic beverages, ready-to-eat meals and cereal and derivatives. Some differences were observed when analyzing food categories per country. In France, the food categories in which potassium additives were present in more than a 50% of labeling were breaded products, non-alcoholic beverages and meat derivatives. In Germany, only meat derivatives had more than a 50% of its labels with any potassium additive. Finally, it should be remarked that in Spain closed to the 92% of breaded products had at least one potassium additive in its formulation, but also the 50% or more of meat derivatives, non-alcoholic beverages, sauces, ready-to-eat meals and fruit/vegetables derivatives labels had any potassium additive.

It should be noted that most of the products analyzed only presented one type of potassium additive among its ingredients. Of the 715 analyzed products, 44 had two potassium additives, 4 products presented three potassium additives and only one, specifically a French meat derivative, included four potassium additives.

**Figure 2:**
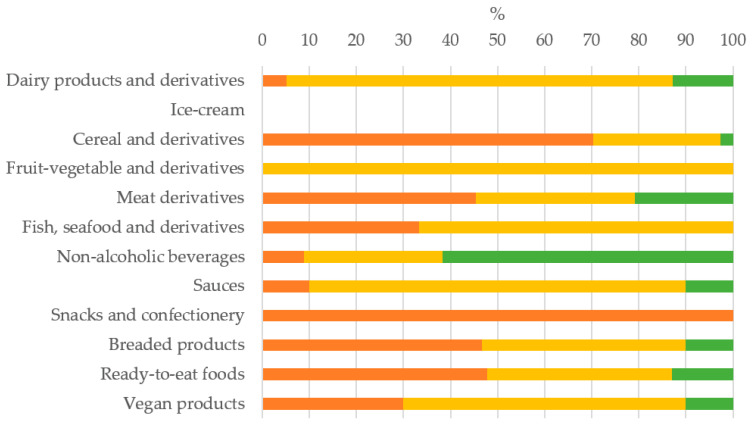
Percentage of each potassium additive group found per food category in the global sample. 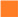 HKC: additives with high potassium content; 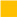 MKC: additives with moderate potassium content; 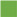 LKC: additives with low potassium content.

Of all the additives found, most belonged to the HKC and MKC group, 36.6% and 45.4%, respectively, and LKC additives only represented the 18%. Per country, it could be observed that HKC + MKC additives represented 90%, 80% and 72.2%, in Spain, France and Germany, respectively. The percentage of each potassium additive group, HKC, MKC or LKC, found per food category in the global sample is shown in Figure 2. Notably, the predominance of the HKC and MKC additives, the most dangerous for CKD patients, was found in all food categories except in non-alcoholic beverage. This pattern remained similar in the three countries. The food categories with the highest proportion of HKC additives were snacks-confectionery and cereal derivatives, while MKC additives predominated in dairy products and derivatives, fruit/vegetable derivatives and sauces.

Table 4 show the number of different types of potassium additives found in each food category. Globally, in the whole sample, the results showed how ready-to-eat meals, breaded products and meat derivatives were the food categories that included the greatest variety of potassium additives. These results were expected, since these food categories has a higher grade of processed or has a higher number of ingredients, some being of them also processed.

**Figure 3:**
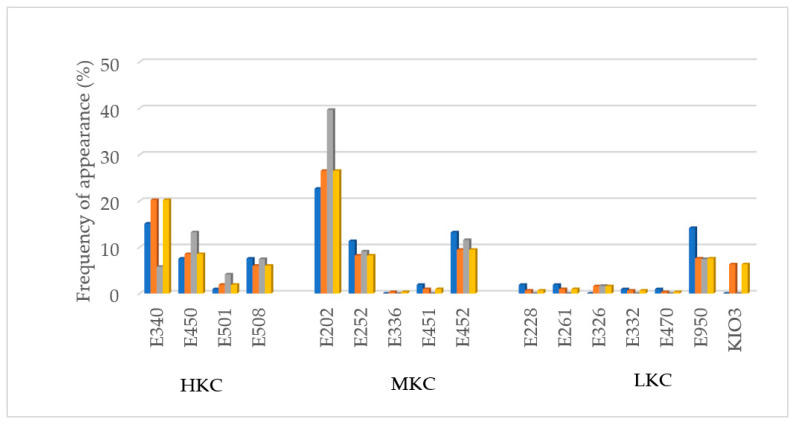
Potassium additives found and frequency of appearance (%) per country and in Europe. HKC: additives with high potassium content; MKC: additives with moderate potassium content; LKC: additives with low potassium content; 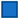 France; 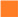 Germany; 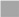 Spain; 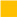 Global sample.

In Figure 3 can be observed the potassium additives found and its appearance frequency. Potassium sorbate (E202) was the additive that appeared most frequently in the foods analyzed, especially in Spain. Secondly, di- and triphosphates (E340, E450) and thirdly polyphosphates (E452). All of them part as HKC and MKC groups. It should be noted that frequently, because it is not mandatory, label only indicated di-, triphosphates or polyphosphates but not its cation, ex. K^+^ or Na^+^. In those cases, the product was considered as possible potassium additive content and included in the list. Additionally, it should be remarked that frequently, the labeling only indicates the general number, (E501) but does not specify the particular compound (ex. E501i or E501ii). This fact implies an additional problem to identify potential dangerous additives in food products. When no specification was indicated regarding additive E501, it was considered has E501ii (HKC) applying a conservative criterion.

In many German products, especially in meat derivatives, it was remarkable the use of potassium iodate (KIO_3_) as flavoring agent salt, instead of sodium or potassium chloride like other countries. Although this compound is not properly an additive because it does not have a technological role in the final product, its inclusion in the analysis is crucial due to its content in potassium.

Additives are used in foods due to its technological and functional properties. Table 5 shows the different uses applied by the additives found in the analyzed foods, their frequency, as well as the particular additives used to achieve this function. It can be observed that the main technological use was as a preservative, due to the high presence of potassium sorbate (E202) and potassium nitrate (E252), followed by their use as stabilizers and flavoring agents. The multi-functionality of the potassium di- and triphosphates group (E340-E450) was especially relevant, since they are used both as stabilizers, raising agents and emulsifying salts, as well as acidity regulators. In addition, these additives belong to the group of those with a high potassium content (HKC) and represent a danger for patients with chronic kidney disease.

Furthermore, it should be noted that in many products potassium chloride (E508) was named in labeling as an ingredient, and not as an additive associated with its technological function, although in most cases, it was used as a flavoring agent to replace common salt. This is probably due to the intention of creating a false consumer perception of less use of additives in the product. However, given the objective of this study, it was counted as presence when it appeared on the label both as a common ingredient and as additive with a technological function.

**Figure 4:**
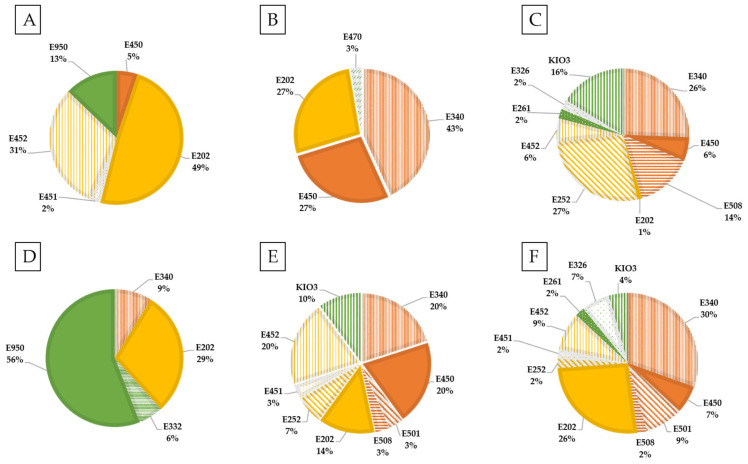
Percentage of each potassium additive by food category. (**A**) Dairy products and derivatives; (**B**) Cereal and derivatives; (**C**) Meat derivatives; (**D**) Non-alcoholic beverages; (**E**) Breaded products; (**F**) Ready-to-eat products.

In Figure 4, the percentage of each potassium additive in the six food categories with the highest proportion of additives containing potassium is represented. Preservatives (E202 and E252) were one of the most numerous additives in almost all food categories, except for non-alcoholic beverage, snacks-confectionery, breaded products, and fish derivatives. In these, sweetener, raising agent and stabilizer were the main technological uses. It should be noted that, in all of the fruit and vegetable derivatives analyzed that contained potassium additives among its ingredients, they only had potassium sorbate (E202) with the preservative function.

In the other categories, such as cereals and derivatives, the additives found were potassium sorbate (E202) as a preservative, and raising agents such as potassium di- and triphosphate (E340-E450). However, in fish derivatives, the addition of potassium phosphates was mainly used as a stabilizer.

It should be remarked that those additives belonging to group I, specifically potassium carbonate (E501) and potassium chloride (E508), which can therefore be added in quantities of *Quantum satis*, were in meat derivatives, breaded products, snacks and ready-to-eat products.

## 4. Discussion

Extensive knowledge of potassium additives, together with food-containing these additives, is crucial to mineral dietary management of CKD patients. This knowledge is also indispensable to prevent hyperkalemia in these patients, as it hints useful and concise information for developing educational tools aimed at these patients.

The results found in this study show that, indeed, potassium from additives is found in a wide variety of processed foods that are currently on the market. Ignorance and lack of control over its presence in foods consumed, could be considered a dangerous hidden source of potassium in the diet and therefore a potential problem for the population suffering from chronic kidney disease, especially those with higher risk of hyperkalemia.

From the results is observed that, although it is true that many additives with potassium in their formulation are currently authorized in Europe, specifically 41, only 16 of them, that is 39%, are used regularly. Cupisti et al. (2018) indicated in their article that the most frequently used potassium additives were 11 in a sample of 25 products [19]. Only 6 of the additives found in the results of this study matched with those indicated by the authors: E202, E252, E332, E336, E450 and E451. This could be because, depending on the food product as well as its origin, manufacturers tend to use different types of additives.

Among the 11 potassium additives found by Cupisti et al. (2018), only one belongs to HKC. However, this study results showed 4. Of the additives found in the labeling analysis, 4 of them were classified as additives with a high potassium content (HKC): di- and tripotassium phosphate (E340), tetrapotassium diphosphate (E450), potassium hydrogen carbonate (E501ii) and potassium chloride (E508). The frequency of appearance of di- and tripotassium phosphate (E340) was especially high. This additive, together with tetrapotassium diphosphate (E450) and potassium polyphosphates (E452), from MKC, consist of potassium salts, and have the relevant characteristic of fulfilling many technological functions, from raising agents and stabilizers to emulsifying salts, which is of interest due to their potential use in many products, depending on the needs of each one. However, it is essential to note that there are counterparts with sodium rather than potassium, which are often indicated as “phosphates” or “polyphosphates”, without defining the particular salt, hindering potassium detection. In addition, the current legislation that regulates food additives [17] allows in most cases to add mixtures of them, and establishing the maximum amounts as mg/L or mg/kg of total P_2_O_5_. All of this, should force to apply a conservative criterion and consider its presence as a possible risk for patients with chronic kidney disease. This type of additives, due to their high phosphorus content, had also aroused concern and alert by various authors previously [10,20,21]. Meat derivatives have demonstrated to be one of the food categories with higher number of potassium additives. The high presence of potassium additives, as well as phosphoric ones, in meat products was previously detected in other works [22]. In this line, the alert about these types of additives had already been included specifically in educational initiatives aimed at kidney patients on additives phosphoric acids in food [23].

Potassium additives with the technological function of preservatives were the most distributed among the foods studied in the present study, being mainly potassium sorbate (E202) from MKC. It is important to note that, although its maximum content in food is regulated by current legislation as explained previously, EFSA has recently revised the limits set for the admissible daily intake (ADI) of some additives, including potassium sorbate (E202). ADI indicates the amount of an additive added to a food that can be ingested on a daily basis, throughout a person’s life, without representing an appreciable risk to health. In 1996, the EFSA expert panel established an ADI value for sorbic acid and potassium sorbate of 25 mg/Kg of weight/day. However, the appearance of new studies with data on toxicity for development and reproduction encouraged, in 2019, the expert panel reduced this ADI value to 11 mg/kg/day for all population groups [24]. However, the ADI values are based on a healthy population, but in the case of CKD patients, tolerance for this type of additives is lower due to their limited excretion of potassium so it could entail more complications for them.

Due to the negative impact of sodium intake in cardiovascular disease and hypertension, there is an increasing number of consumers demanding foods with a lower salt content (specifically sodium chloride), as well as many institutional initiatives in this line has been promulgated in the last decade [25], are forcing many manufacturers to reformulate their products. According to Regulation No. 1169/2011 about the information provided to consumers in labelling [26], there is currently an obligation to include the salt content, calculated as grams of sodium × 2.5, on the nutritional information reflected on the labeling. For these, they have opted for two alternatives, either reducing the total sodium chloride added in food manufacturing or replacing it with potassium chloride (E508) as a flavor enhancer. By substituting sodium chloride for potassium chloride, as has been observed in the results of the label analysis in some low-salt meat derivatives, the manufacturer manages to lower the salt content of its labeling without sacrificing taste. However, this could be a serious problem for CKD patients, since that same legislation does not express the obligation to indicate food potassium content in the label, and since it is an additive that can be added in quantities of *Quantum satis*, the patient has no way to know the final potassium content of the food product.

A recent study carried out in Canada by Parpia et al. (2018) evaluated the potassium content from additives in low-sodium meat foods. Their results showed that 63% of the products that included the “Low Sodium” claim contained additives with potassium in their ingredient list, but also 26% of those that did not include such a claim contained them, something that has also been observed in this work [27]. The authors observed that low-sodium products contained 44% more potassium than those not classified as low-sodium, and that the average potassium content in these products ranged from 210 to 1500 mg/100 g [27], which could be up to 75% of the recommended daily amount of potassium for patients with CKD. It is important to note that the additives found in low-sodium products were potassium chloride, potassium lactate and potassium phosphate, all of them also found in the present study for European food products. Although for non-low-sodium products, manufacturers currently tend to use the sodium form rather than the potassium form of other additives such as nitrates (E251) and nitrites (E250), this could change over time due to the trend to reduce the salt content of foods as indicated above.

Like phosphorous and potassium additives, the high presence of sodium in additives added in processed products should be a concern in the dietary management of patients with CKD. Reducing salt intake has also been demonstrated as crucial in CKD management to reduce risk factors for heart disease and to slow disease progression in people with CKD in the short term [28,29,30]. Intake of this mineral should not exceed 2 g of sodium per day (or <90 mmol of sodium per day, or <5 g of sodium chloride per day) according to latest KDIGO guidelines [31]. As observed with the other minerals, a more precise and detailed analysis of sodium additives in food products should be done to enhance the dietary guidelines and education tools for CKD patients.

Likewise, it is important to note that, based on the results of the study, many of the additives with potassium have been found in breaded and ready-to-eat products, which usually contain a long list of ingredients. This could be a serious problem according to the indication included in Article 20 of Regulation No. 1169/2011 [26]. The current legislation allows the omission of constituents of food from the ingredients list, specifically food additives, whose presence in a given food is solely due to the fact that they were contained in one or more ingredients of that food, when these no longer fulfill a technological function in the finished product or if they are used as processing aids. That implies, some of the ingredients may contain additives with potassium, and yet not appear on the labeling of the final product. From our label analysis, we suspect that many manufacturers of ready-to-eat products has applied what is stated in this article as no additives were found on the label but its ingredients have a high probability of containing them (ex. meat lasagna). This poses a serious difficulty in identifying potentially dangerous foods for CKD patients. Previous studies have already indicated the problems that the high use of additives, specifically those containing phosphorus, like fast food products, many of them pre-cooked and breaded, imply for patients with kidney disease [32]. This study has shown that not only phosphorous additives present in this type of food are a problem, but also potassium additives.

On the other hand, the presence of acesulfame K (E950) as a sweetener in foods, especially non-alcoholic beverages, should be cause for reflection. Although acesulfame K was classified as one of the additives with low potassium content (LKC), it was the one with the highest presence in this type of beverages (7.6%). The maximum allowed dose of this additive in this type of product is 350 mg/L [16], that is, 115.5 mg/can. But since acesulfame K is a synthetic sweetener with a sweetening power between 100–150 times higher than sucrose, it is foreseeable it will be used in smaller amounts than the maximum allowed dose. In addition to this, and although the supply of soft drinks low-sugar and sugar-free with sweeteners has expanded in recent years, which could imply a problem, the consumption data of soft drinks tends to reduce in last years. With the exception of acesulfame-K, the frequency of appearance of the rest of LKC additives was low. Therefore, it can be concluded that they do not pose a particularly high risk for patients with chronic kidney disease.

On the one hand, following the example of other non-EU countries where it is assessed, the results obtained support the request to include in European legislation the potassium content information on food labeling as mandatory as an aid for vulnerable patients, like it is now salt-sodium content for hypertension control. On the other hand, this study could serve as a tool for health professionals who deal with CKD patients, when approaching a preventive nutritional education program against hyperkalemia. The results allow one to know which additives may pose a greater risk for this population group and that therefore they should try to limit themselves as much as possible, as well as in which processed foods of those currently on the European market there is a greater probability of appearance.

The results of this study allow an overview of the problems related to potassium additives faced by patients with chronic kidney disease. However, it presents a series of limitations that must also be taken into account. On the one hand, it is a descriptive cross-sectional study carried out on products that are currently marketed in only three European countries. As already indicated, although the legislation that regulates the use of food additives is common throughout the European Union, and these three countries allow to get a global idea of the problem, could be some differences in preferences between the use of one type of additive or another between countries, as it has been demonstrated in this work with the preference of KIO_3_ use instead of common salt (NaCl) in German products. So that it would be interesting the food market analysis of other countries, as well as about other types of additives with negative implication in CKD dietary management, like sodium additives. On the other hand, it is likely that consumer demand for products with a lower salt content will force producers to reformulate their products, and probably there will be some change in the type and quantity of additives used in the processed products in the future.

Lastly, the study made it possible to estimate the frequency of occurrence of potassium additives that could pose a problem for patients with chronic kidney disease, but the fact that it was not mandatory to indicate on the label the amount of additive used or the final content of potassium difficult a more precise estimation of the real risk of each product. Therefore, a future next work would be to evaluate the total potassium content, through chemical analysis, of the selected foods with and without the presence of additives, which would allow a comparison of the average contents of this mineral and the effect that the use of additives has on him.

## 5. Conclusions

In the current European legislation about the use of additives in food products, 41 additives containing potassium in their chemical formulation were identified. Of them, 18 belonged to LKC, 15 to MKC, and 8 to HKC. The latter are the ones that pose the greatest danger to patients with chronic kidney disease. To evaluate the risk that a potassium additive may pose to these patients, it is necessary, together with the relative weight of potassium in the chemical formula of the additive, know the maximum additive amount allowed in each type of food product. Of the 41 authorized potassium additives, 16 of them can be added in quantities of *Quantum satis*, 4 of them being part of HKC, the highest risk group: potassium carbonate (E501), potassium chloride (E508), potassium sulfate (E515) and potassium hydroxide (E525). Other additives that should be taken into account by this population, either due to high potassium content or because of their widespread authorized use in processed foods, are potassium nitrite (E249), potassium nitrate (E252), potassium sorbate (E202), di and tripotassium phosphates (E340), and tetrapotassium diphosphate (E450). The analysis of the labeling of 715 products from different food categories revealed that almost 37.6% of the processed products contained some additive with potassium among their ingredients, although important differences between the three countries studied were observed. The most frequent additives found were potassium sorbate (E202), potassium di- and triphosphates (E340), tetrapotassium diphosphate (E450), potassium polyphosphates (E452), and potassium chloride (E508), being all of them part of the HKC and MKC groups. These additives belong to the high and medium potassium groups (HKC and MKC), however, the maximum amount that can be added to food is regulated for all of them except for E508, which can be added in *Quantum satis.* The main technological uses of these additives in food were as preservatives, stabilizers, and flavoring agent. The food categories that showed the greatest presence of additives were breaded products, meat derivatives, non-alcoholic beverage, ready-to-eat products and cereal derivatives. On the contrary, those that showed the least presence were ice creams, fish derivatives, foods for vegetarians and snack-confectionery products. Although dairy products, fruit/vegetable derivatives, sauces and snack-confectionery products presented low appearance frequency of potassium additives, if they were present belonged to HKC and MKC, so that is also important to control them through its labelling. Similar problematic situation was observed in cereal derivatives category, in which the main part of potassium additives found were HKC. Potassium additives are widely distributed in processed foods and therefore pose a risk of hidden sources of potassium in CKD dietary management for hyperkalemia prevention. The lack of information on the food labeling on potassium content limited the provision of more precise information on the total potassium content in the food. This problem, also present in the United States and Canada, was addressed by the authorities, and currently, the legislation on labeling of food products requires the inclusion of information on the content of certain minerals such as potassium on labels. In Europe, given that this aspect is not contemplated in current legislation, the authorities should be urged to implement it as well.

## Figures and Tables

**Table 1:** Food categories and sampling of each category per country.

Food Category	France (*n* = 270)	Germany (*n* = 233)	Spain (*n* = 212)	Total (*n* = 715)
Dairy products and derivatives	65	34	33	132
Ice-cream	4	3	3	10
Cereal and derivatives	29	24	23	76
Fruit/vegetable and derivatives	14	12	12	38
Meat derivatives	39	37	27	103
Fish, seafood and derivatives	6	6	6	18
Non-alcoholic beverages	19	18	15	52
Sauces	21	18	18	57
Snacks and confectionery	12	15	15	42
Breaded products	15	12	12	39
Ready-to-eat foods	28	33	27	88
Vegan products	18	21	21	60

**Table 2:** Authorized food additives with potassium classification according to its chemical formulation and potassium representative weight.

LKC * (<25% by Weight of K)			MKC * (25–39% by Weight of K)			HKC * (≥40% by Weight of K)		
E-Number	Name	Potassium Weight (%)	E-Number	Name	Potassium Weight (%)	E-Number	Name	Potassium Weight (%)
E 212	Potassium benzoate	18.1	E 202	Potassium sorbate	25.8	E 249	Potassium nitrite	43.6
E 228	Potassium hydrogen sulphite	9.1	E 224	Potassium metabisulphite	31.7	E 340	Potassium diphosphates	44.0
E 261	Potassium acetate	39.4	E 252	Potassium nitrate	38.3	E 340	Potassium triphosphates	53.6
E 261	Potassium diacetate	15.8	E 283	Potassium propionate	34.5	E 450	Tetrapotassium diphosphate	45.0
E 326	Potassium lactate	20.1	E 332	Potassium citrates	35.8	E 501ii	Potassium hydrogen carbonate	56.0
E 332	Potassium citrates	16.8	E 336	Potassium ditartrates	32.9	E 508	Potassium chloride	51.9
E 336	Potassium tartrates	16.8	E 340	Potassium monophosphates	28.4	E 515	Potassium sulphates	44.4
E 337	Sodium potassium tartrate	13.7	E 351	Potassium malate	26.0	E 525	Potassium hydroxide	62.7
E 402	Potassium alginate	17.4	E 357	Potassium adipate	34.8			
E 470a	Sodium, potassium and calcium salts of fatty acids	17.9	E 451ii	Pentapotassium triphosphate	39.2			
E 522	Aluminium potassium sulphate	8.2	E452ii	Potassium polyphosphate	32.8			
E 555	Potassium aluminium silicate	9.7	E 456	Potassium polyaspartate	25.0			
E 577	Potassium gluconate	16.5	E 501	Potassium carbonates	38.7			
E 622	Monopotassium glutamate	19.0	E 515i	Potassium acid sulfate	28.4			
E 628	Dipotassium guanylate	17.3	E 536	Potassium ferrocyanide	36.7			
E 632	Dipotassium inosinate	17.9						
E 950	Acesulfame K	19.2						
E 954	Saccharin and its Na, K and Ca salts	16.1						

* LKC: additives with low potassium content; MKC: additives with moderate potassium content; HKC: additives with high potassium content.

**Table 3:** Food categories included in Group I according Commission Regulation (EU) No. 1129/2011 [16], in which additives may be included in amounts of *Quantum satis*.

Food Category	Specifications
Dairy products	Unflavored fermented dairy products heat-treated after fermentation
	Flavored fermented dairy products, even heat-treated
	Other creams
	Unripened cheese, except mozzarella and unripened cheese fermented by the action of living organisms, without flavoring
	Processed cheese
	Cheese products (excluding dessert products)
	Dairy analogues, including beverage whiteners
Ice-cream	Ice-cream
Fats, oils and their emulsions	Other fat and oil emulsions including spreads as defined by Regulation (EC) No 1234/2007 and liquid emulsions
	Vegetable oil pan spray
Fruits and vegetables	Dried fruit and vegetables
	Fruit and vegetables in vinegar, oil, or brine
	Fruit and vegetable preparations excluding compote
	Nut butters and nut spreads
	Processed potato products
Nuts	Processed nuts
Desserts	Desserts excluding dairy products, ice-creams, and fruit vegetables.
Confectionery products	Cocoa and chocolate products, energy-reduced only or no added sugar
	Other confectionery products, including breath fresheners
	Chewing gum
	Decorations, coatings and fillings
Cereals and derivatives	Starches
	Breakfast cereals
	Dry pasta, gluten-free or intended for low-protein diets
	Potato gnocchi
	Fillings of stuffed pasta (ravioli and similar)
	Noodles
	Batters
	Pre-cooked or processed cereals
	Bread and rolls excluding those prepared solely with the following ingredients: wheat flour, water, yeast or leaven, salt.
	Fine bakery wares
Ready-to-eat savouries and snacks	Potato-, cereal-, flour- or starch-based snacks
Meat and meat derivatives	Non-heat-treated processed meat
	Heat-treated processed meat *excluding foie gras, foie gras entier, blocs de foie gras.*
	Casings and coatings and decorations for meat
Seafood and derivatives	Processed fish and fishery products including mollusks and crustaceans
	Fish roe
Eggs and egg products	Processed eggs and egg products
Salt, spices, soups, sauces, salads, and protein products	Other sugars and syrups
	Salt Substitutes, Seasonings, and Dressings
	Sauces
	Vinegars
	Mustard
	Soups and broths
	Salads and savoury based sandwich spreads
Beverages	Fruit juices and vegetable or legume juices
	Fruit nectars
	Flavoured drinks
	Others
	Cider and perry
	Fruit wine and made wine
	Mead
	Spirit drinks
	Aromatised wines
	Aromatised wine-based drinks
	Aromatised wine-product cocktails
	Other alcoholic drinks including mixtures of alcoholic drinks with non-alcoholic drinks and spirits with less than 15% of alcohol
Foods intended for particular nutritional uses as defined by Directive 2009/39/EC.	Dietary foods for special medical purposes
	Dietary foods for weight control diets intended to replace total daily food intake or an individual meal (the whole or part of the total daily diet)
	Foods suitable for people intolerant to gluten
Food supplements	

**Table 4:** Number of different types of potassium additives found per food category.

Food Category	France	Germany	Spain	Global Sample
Dairy products and derivatives	3	3	4	5
Ice-cream	0	0	0	0
Cereal and derivatives	4	3	3	4
Fruit/vegetable and derivatives	1	0	1	1
Meat derivatives	6	6	7	9
Fish, seafood and derivatives	0	0	2	2
Non-alcoholic beverages	3	2	3	4
Sauces	2	2	2	4
Snacks and confectionery	1	1	2	2
Breaded products	5	2	6	9
Ready-to-eat foods	6	6	5	11
Vegan products	2	2	2	4

**Table 5:** Main functional properties of potassium additives found in Europe and the technological use of potassium additives frequency.

Functional Property	Frequency of Use (%)	Main Potassium Additives Used
Preservative	36.96	E202/E252/E261
Stabilizer	20.19	E340/E450
Flavoring agent	12.73	E508/KIO3
Sweetener	10.56	E950
Emulsifying salt	7.45	E340/E450/E451/E452
Raising agent	6.52	E340/E450/
Acidity regulators	3.73	E332/E501/E326/E340/E452
Other	1.86	

## Appendix A

**Table A1:** Food category and maximum level permitted of additive group E338-452: Phosphoric acid, phosphates, di-, tri- and polyphosphates.

Food Category	Specifications	Maximum Level (mg/L or mg/Kg)
Dairy products	Unflavoured sterilized and UHT milk	1000
	Flavoured fermented milk products including heat-treated products	3000
	Partly dehydrated milk with less than 28% solids	1000
	Partly dehydrated milk with more than 28% solids	1500
	Dried milk and dried skimmed milk	2500
	sterilized, pasteurized, UHT cream and whipped cream	5000
	Unripened cheese except mozzarella	2000
	Processed cheese	20,000
	Unripened cheese products	2000
	Whipped cream analogues	5000
	Processed cheese analogues	20,000
	Beverage whiteners	30,000
Fat and oil emulsions	Soured cream butter	2000
	Spreadable fats	5000
Ice-cream		1000
Fruit and vegetables	Fruit preparations excluding compote	800
	Glazings for vegetable products	4000
	Nut butters and nut spreads (spreadable fats excluding butter)	5000
	Processed potato products including pre-fried frozen en deep-frozen potatoes	5000
Desserts	Desserts	3000
	Dry powdered dessert mixes	7000
Confectionery	Sugar confectionery, except candied fruit	5000
	Candied fruit	800
	Chewing gum	*Quantum satis*
	Decorations, coatings and fillings, except fruit-based fillings	5000
	Toppings (syrups for pancakes, flavoured syrups for milkshakes and ice cream; similar products)	3000
Cereals and cereal products	Flours	2500
	Self-raising flour	20,000
	Breakfast cereals	5000
	Noodles	2000
	Batters	12,000
	Soda bread and fine bakery wares	20,000
Ready-to-eat savouries and snacks	Potato-, cereal-, flour- or starch-based snacks	5000
Meat and meat preparations	Non-heat-treated processed meat	5000
	Heat-treated processed meat (except foie gras)	5000
	Glazings for meat	4000
Fish and fisheries products	Frozen and deep-frozen fish fillets, molluscs and crustaceans	5000
	Canned crustaceans products; surimi and similar products	1000
	Fish and crustacean paste and in processed frozen and deep-frozen molluscs and crustaceans	5000
Eggs and egg products	Liquid egg (white, yolk or whole egg)	10,000
Salts, spices, soups, sauces, salads and protein products	Salt and salt substitutes	10,000
	Soups and broths	3000
	Sauces	5000
	Vegetable protein drinks	20,000
Alcoholic and non-alcoholic beverages	Water, including natural mineral water	500
	Flavored drinks	700
	Whey protein containing sport drinks	4000
	Vegetable protein drinks	20,000
	Chocolate and malt dairy-based drinks	2000
	Coffee-based drinks for vending machines; Instant tea and instant herbal infusions	2000
	Cider and perry	1000
	Fruit wine and made wine	1000
	Mead	1000
	Spirit drinks (except: whisky, whiskey)	1000
	Aromatized wines	1000
	Aromatized wine-based drinks	1000
	Other alcoholic drinks including mixtures of alcoholic drinks with non-alcoholic drinks and spirits with less than 15% of alcohol	1000
Foods intended for particular nutritional uses	Infant formulae as defined by Directive 2006/141/EC	1000
	Follow-on formulae as defined by Directive 2006/141/EC	1000
	Processed cereal-based foods and baby foods for infants and young children as defined by Directive 2006/125/EC	1000 (sonly cereals-E340) 5000 (biscuits and rusks-E450)
	Other foods for young children	1000
	Dietary foods for infants for special medical purposes and special formulae for infants	1000
	Foods suitable for people intolerant to gluten	5000
	Dietary foods for weight control diets intended to replace total daily food intake or an individual meal (the whole or part of the total daily diet)	5000
Food supplements		*Quantum satis*

**Table A2:** Food category and maximum level permitted of additive potassium sorbate (E202).

Food Category	Specifications	Maximum Level (mg/L or mg/Kg)
Dairy products	Curdled milk	1000 *
	Non-heat-treated dairy-based desserts	300 **
	Unripened cheese	1000 *
	Cheese, prepacked, sliced and cut; layered cheese and cheese with added foods	1000 *
	Ripened products surface treatment	*Quantum satis*
	Whey cheese, prepacked, sliced; layered cheese and cheese and cheese with added foods	1000 *
	Processed cheese	2000 *
	Unripened products; ripened products, prepacked, sliced; layered ripened products and ripened products with added foods	1000 *
	Analogues of cheese based on protein	2000
	Ripened products surface treatment	*Quantum satis*
Fat and oil emulsions	Fat emulsions (excluding butter) with a fat content of 60% or more	1000 *
	Fat emulsions with a fat content less than 60%	2000 *
Fruit and vegetables	Surface treatment of unpeeled fresh citrus fruit	20 *
	Dried fruit	1000 *
	Olives and olive-based preparations	1000 *
	Vegetables in vinegar, oil, or brine (excluding olives)	2000 **
	Fruit and vegetable preparations including seaweed based preparations, fruit-based sauces, aspic, excluding purée, mousse, compote, salads and similar products, canned or bottled	1000 *
		1000 ** (olive-based preparations)
	Low-sugar and similar low calorie or sugar-free products, mermeladas	1000 **
	Low-sugar and similar low calorie or sugar-free products, spreads, mermeladas	1000 **
	Fruit-based spreads, mermeladas	1000 **
	Marmelade	1500 **
	Potato dough and pre-fried potato slices	2000
Nuts	Coated nuts	1000 ***
Desserts	Non-heat-treated dairy-based desserts	300 **
Confectionery	Candied, crystallized or glacé fruit and vegetables	1000 **
	Other confectionery including breath freshening microsweets	1500 ***
	Chewing gum	1500 **
	Toppings (syrups for pancakes, flavored syrups for milkshakes and ice cream; similar products)	1000
	Other decorations, coatings and fillings, except fruit-based fillings	1500 ***
Cereals and cereal products	Potato Gnocchi	1000
	Fillings of stuffed pasta (ravioli and similar)	1000
	Batters	2000 *
	Polenta	200 *
	Prepacked sliced bread and rye-bread, partially baked, prepacked bakery wares intended for retail sale and energy-reduced bread intended for retail sale	2000
	Fine bakery wares (only with Aw >0.65)	2000
Ready-to-eat savouries and snacks	Potato-, cereal-, flour- or starch-based snacks	1000 **
Meat and meat preparations	Surface treatment of dried meat products	*Quantum satis ****
	Pâté	1000 ***
	Collagen-based casings with Aw >0.6	*Quantum satis*
	Jelly coatings of meat products (cooked, cured or dried)	1000 ***
Fish and fisheries products	Salted, dried fish	200
	Semi-preserved fish and fisheries products including crustaceans, molluscs, surimi and fish/crustacean paste; cooked crustaceans and molluscs	2000 **
	Semi-preserved fish products including fish roe products	2000 **

* The additives may be added individually or in combination. The maximum level is applicable to the sum and the levels are expressed as the free acid. ** The additives may be added individually or in combination with sorbic acid, potassium sorbate, benzoic acid and benzoates. The maximum level is applicable to the sum and the levels are expressed as the free acid. *** The additives may be added individually or in combination with sorbic acid, potassium sorbate, benzoic acid and benzoates and E214-219: p-hydroxybenzoates (PHB) (maximum 300 mg/kg). The maximum level is applicable to the sum and the levels are expressed as the free acid.

**Table A3:** Food category and maximum level permitted of additive potassium nitrate (E252).

Food Category	Specifications	Maximum Level (mg/L or mg/Kg)
Meat and meat preparations	Non-heat-treated processed meat	150 *
	Traditional immersion and dry cured products (Meat products cured by immersion in a curing solution containing nitrites and/or nitrates, salt and other components) or Traditional dry cured products. (Dry curing process involves dry application of curing mixture containing nitrites and/or nitrates, salt and other components to the surface of the meat followed by a period of stabilization/maturation).	250 **
Dairy products	Ripened cheese (hard, semi-hard and semi-soft cheese)	150
	Whey cheese (cheese milk of hard, semi-hard and semi-soft cheese)	150
	Cheese products (hard, semi-hard and semi-soft ripened products)	150
	Dairy analogues, including beverage whiteners (only dairy-based cheese analogue)	150
Fish and fisheries products	Processed fish and fishery products including molluscs and crustaceans (only pickled herring and sprat)	500

* Maximum added amount ** Maximum residual amount, residue level at the end the production process.
